# Identification and Expression Profile of Olfactory Receptor Genes Based on *Apriona germari* (Hope) Antennal Transcriptome

**DOI:** 10.3389/fphys.2020.00807

**Published:** 2020-07-22

**Authors:** Jia-Li Qian, Ding-Ze Mang, Guo-Chang Lv, Jia Ye, Zhao-Qun Li, Bo Chu, Long Sun, Yu-Jun Liu, Long-Wa Zhang

**Affiliations:** ^1^Anhui Provincial Key Laboratory of Microbial Control, Engineering Research Center of Fungal Biotechnology, Ministry of Education, School of Forestry and Landscape Architecture, Anhui Agricultural University, Hefei, China; ^2^Graduate School of Bio-Applications and Systems Engineering, Tokyo University of Agriculture and Technology, Tokyo, Japan; ^3^Tea Research Institute, Chinese Academy of Agricultural Sciences, Hangzhou, China; ^4^Anhui Academy of Science and Technology, Hefei, China

**Keywords:** odorant receptor, ionotropic receptor, expression pattern, antennal transcriptome, *Apriona germari*

## Abstract

Insects’ olfactory receptor plays a central role in detecting chemosensory information from the environment. Odorant receptors (ORs) and ionotropic receptors (IRs) are two types of olfactory receptors, and they are essential for the recognition of ligands at peripheral neurons. *Apriona germari* (Hope) (Coleoptera: Cerambycidae) is one of the most serious insect pests that cause damage to economic trees and landscaping trees, resulting in massive environmental damages and economic losses. Olfactory-based management strategy has been suggested as a promising strategy to control this wood-boring beetle. However, the olfactory perception mechanism in *A. germari* is now almost unknown. In the present study, RNA sequencing analysis was used to determine the transcriptomes of adult *A. germari* antennae. Among 36,834 unigenes derived from the antennal assembly, we identified 42 AgerORs and three AgerIRs. Based on the tissue expression pattern analysis, 27 AgerORs displayed a female-biased expression. Notably, AgerOR3, 5, 13, 33, and 40 showed a significant female-biased expression and were clustered with the pheromone receptors of *Megacyllene caryae* in the phylogenetic tree, suggesting that these AgerORs could be potential pheromone receptors for sensing male-produced sex pheromones in *A. germari*. The AgerIRs expression profile demonstrated that AgerIR2 had high expression levels in male labial palps, suggesting that this receptor may function to detect female-deposited trail-sex pheromone blend of *A. germari*. In addition, the phylogenetic tree showed that the Orco gene of five cerambycidae species was highly conservative. These results provide a foundation for further studies on the molecular mechanisms of olfactory chemoreception in *A. germari* apart from suggesting novel targets for the control of this pest in the future.

## Introduction

Insects have developed a set of highly specialized and sensitive olfactory system that can accurately identify miniscule and highly specific odor substances in a complex natural environment and perform adaptive behaviors by long-term selective evolution (Jacquin-Joly and Merlin, 2005; Martin et al., 2011). The accurate recognition of chemical signals in insects is mainly accomplished by the bristles of sensory taste and smell neurons on their cuticles, among which the olfactory sensilla are mainly distributed in the antennae and the maxillary palp of insects. It is thought that olfactory recognition in insects is a complex process involving multiple chemosensory-associated proteins, including chemosensory proteins, odorant receptors (ORs), odorant-binding proteins, odorant-degrading enzymes, ionotropic receptors (IRs), and sensory neuron membrane proteins (Sato and Touhara, 2009; Leal, 2013). The initial steps in odor detection involve the binding of an odor to the OR displayed on the dendrites of olfactory sensory neurons, which leads to signal transduction (chemical signals become electrical signals), before they are transmitted to the central nervous system for complete odor perception (Vogt and Riddiford, 1981; Durand et al., 2011; Bohbot and Dickens, 2012). Therefore, olfactory receptors are one of the key components of the olfactory system (Gong et al., 2008; Zhan et al., 2018). Since the discovery of olfactory receptors in *Drosophila melanogaster* in 1999, the olfactory receptor system has been a key link in understanding the molecular recognition mechanism of insects’ chemical signals. This also provides the basis for the specific control of agricultural and forestry pests through insect smell.

The olfactory receptors of insects include ORs and IRs. ORs are composed of about 400 amino acids and has seven transmembrane domains, with the N-terminal on the inner side of the cell and C terminus on the outer side of the cell, in contrast to the G-protein coupled receptor family (Benton et al., 2006; Lundin et al., 2007). The OR of insects can be divided into two categories: one is typical odor receptor, which includes common odor receptors and sex pheromone receptors representing the majority of ORs, which are lowly conserved between subspecies; the other is atypical odor receptor (Orco) which comprises a small number of proteins, highly conserved between subspecies, but is not directly involved in the perception of odor molecules (Nichols and Luetje, 2010; Nichols et al., 2011; Wang et al., 2018). ORs mainly recognize odorants like volatiles and less volatile matter, including plant volatiles, fragrance material, herbivore-induced plant volatiles, sex pheromones, and intraspecific volatiles such as alarm pheromones and aggregation pheromones (Dweck et al., 2013; Cattaneo et al., 2017; Zhang et al., 2017; Wu et al., 2018). IRs are olfactory receptors identified by Benton in the antennae of fruit flies, belonging to the ionotropic glutamate receptor family (Benton et al., 2009). IRs and ORs play a complementary role in odor recognition (Abuin et al., 2011), although IRs and ORs generally recognize different odor substances. ORN-expressing IRs are less sensitive to odor substances than those of ORN-expressing ORs, and the electrophysiological response rate of IR to odor substances is also slower than that of OR (Yao et al., 2005; Getahun et al., 2012). Functional studies of IRs in *D. melanogaster* have shown that they play an important role in the perception of the environment, such as smell, taste, humidity, and temperature (Hussain et al., 2016; Chen and Amrein, 2017; Knecht et al., 2017; Prieto-Godino et al., 2017; Budelli et al., 2019).

The brown mulberry longhorn beetle, *Apriona germari* (Hope) (Coleoptera: Cerambyciade, Lamiinae), native to China, Japan, North Korea, Thailand, and India, is one of the most serious insect pests that cause damage to economic trees and landscaping trees (Huang, 1999; Bi et al., 2017). It is a polyphagous xylophage that mainly damages *Populus* spp., *Morus alba* L., *Broussonetia papyrifera* L., *Ulmus pumila* L., *Malus pumila* Mill., *Cerasusus pseudocerasus* G., *Pyrus* spp., and *Citrus reticulata* (Huang, 1999; Zhang et al., 2011). In recent years, many provinces in China have vigorously promoted the program of returning farmland to forests, and as a result, areas of *Populus* spp. and *Morus alba* L. have expanded rapidly. However, damage caused by wood-boring beetles occurs almost every year. Among them, *A. germari* has been listed as a “second-level” harmful forest pest, with an affected area of over 666.67 km^2^, threatening the consolidation of afforestation efforts, economic benefits, and ecological effects (Lu et al., 2001; Tang et al., 2013). Given the devastating damage of *A. germari* to forests, an efficient and sustainable control tool still remains a challenge. Olfactory-based management strategy has been considered as a promising strategy to control this wood-boring beetle. However, olfactory perception mechanisms in *A. germari* are currently almost unknown. In order to increase our understanding of the olfactory receptor genes involved in this beetle, we carried out our studies by focusing on (1) analyzing the transcriptome data of adult *A. germari* antennae using bioinformatics, followed by screening of and identifying olfactory receptor genes, (2) examining the expression of olfactory receptor genes of both male and female adults using quantitative real-time polymerase chain reaction (qRT-PCR), and (3) providing valuable information for analyzing the role of olfactory receptor genes involved in the molecular chemoreception mechanisms of olfaction in *A. germari*.

## Materials and Methods

### Insect and Tissue Collections

The newly emerged adults of *A. germari* were collected from a poplar plantation in Bengbu Forest Farm, Anhui Province, China, in June 2018. The branches of *B. papyrifera* were collected in a centrifugal tube (50 ml) with a temperature of 25 ± 1°C and humidity of 65 ± 5%. Vigorous male and female adults were selected, and their antennae, maxillary palps, labial palps, and abdominal parts were immediately snap-frozen in liquid nitrogen and stored separately at −80°C until use.

### RNA Extraction, cDNA Library Construction, and Next-Generation Sequencing

Total RNA was extracted from *A. germari* adults’ antennae (both male and female) by TRIzol method. The RNA samples were sent to Novogene Co., Ltd. (Beijing, China) for non-reference transcriptome sequencing. cDNA library construction and next-generation sequencing were performed as previously described (Sun et al., 2018). In brief, RNA purity was checked using a NanoPhotometer spectrophotometer (IMPLEN, CA, United States). NEBNext^®^ Ultra^TM^ RNA Library Prep Kit (NEB, United States) was used to generate sequencing libraries, and index codes were added to attribute sequences to each sample. The cBot Cluster Generation System was used to generate a cluster of the index-coded samples with TruSeq PE Cluster Kit v3-cBot-HS (Illumia, San Diego, CA, United States), following the manufacturer’s instructions. After cluster generation, an Illumina Hiseq platform was used to sequence the library preparations and generate paired-end reads.

### Assembly and Functional Annotation

Transcriptome assembly was accomplished based on clean reads using Trinity (V2.4.0) to generate transcripts (Grabherr et al., 2011). The clean reads obtained by sequencing were spliced to obtain transcripts, and the longest transcript of each gene was selected as Unigene. The transcript sequence was compared with protein databases NCBI (non-redundant, Nr), Swiss-Prot, Kyoto Encyclopedia of Genes and Genomes (KEGG), and Clusters of Orthologous Groups of proteins (*E*-value < 10^–5^) by Blastx and nucleic acid databases Nt (*E*-value < 10^–5^) by Blastn. The transcript was annotated as the protein with the highest consistency. Open reading frame (ORF) finder^1^ was used to find ORFs for related genes. The transmembrane prediction of receptor genes is based on TMHMM Server v. 2.0^2^ online software. All nucleic acid sequences were translated into amino acid sequences by Primer Premier 5 software.

### Phylogenetic Analysis

The amino acid sequences of candidate receptor genes were aligned by BioEdit with similar reported species. Then, MEGA5 was used to make phylogenetic trees by neighbor-joining method and bootstrap with 1,000 replicates (Saitou and Nei, 1987). The evolutionary tree of candidate ORs was constructed from the protein sequences of *Anoplophora chinensis* (Sun et al., 2018), *Anoplophora glabripennis* (Mitchell et al., 2017), *Agrilus planipennis* (Mitchell et al., 2020), *Dendroctonus ponderosae* (Andersson et al., 2013), *Tribolium castaneum* (Engsontia et al., 2008), *Anomala corpulenta* (Li et al., 2015), *Monochamus alternates* (Wang et al., 2014), *Megacyllene caryae* (Mitchell et al., 2012), and *Tenebrio molitor* (Liu et al., 2015). The evolutionary tree of candidate IRs was built with the aligned protein sequences from *A. chinensis*, *I. typographus*, *A. glabripennis*, *D. ponderosae*, *D*. *melanogaster* (Benton et al., 2009), *Phyllotreta striolata* (Wu et al., 2016), *T. castaneum* (Croset et al., 2010), *A. corpulenta*, *M. alternates*, and *T. molitor*.

### qRT-PCR Validation of ORs and IRs

Total RNA was isolated from 30 antennae, 80 maxillary palps, 80 labial palps, and 30 body ends from each sex. Isolated RNA was reverse-transcribed into cDNA using PrimeScriptTMRT reagent kit with gDNA Eraser (Perfect Real Time, TaKaRa, Beijing, China). qRT-PCR validation was carried out as described in Sun et al. (2018) but using different primers (Supplementary Table S1). Three biological replications were carried out for each sample and measured in three technique replications. The variability of each gene expression in different tissues was confirmed by using Q-Gene method (Simon, 2003). Graphical plot mapping was done by GraphPad prism v5.0 Software (GraphPad Software Inc., CA, United States). The relative expression of mRNA of each gene (mean ± SD) was analyzed using one-way ANOVA (SPSS22.0 for Windows, IBM, United States), followed by Duncan’s new multiple-range test (*a* = 0.05).

## Results

### Transcriptome Sequencing and Homology Assembly

The transcriptome information of the longicorn beetle, *A. germari*, was characterized by constructing a cDNA library prepared from purified mRNA isolated from the adults’ antennae. HiSeq 2500 system generated a total of 49,729,910 raw reads and 47,631,128 clean reads. The Q20 and Q30 base call accuracies were 97.79 and 94.26%, respectively (Table 1). From these, 62,299 unigenes were screened from 115,952 transcripts. The mean length of the transcript and the unigene was 647 and 948 bp, respectively, with an N50 of 1,007 and 1,295 bp, respectively (Table 1). The length frequency distribution for unigenes and transcripts (Supplementary Figure S1) showed declines in the number of transcripts with increasing length. However, the number of unigenes was increased at first and then declined in the range between 1,001 and 2,000 bp. Less than one-fifth of short reads (<301 bp) were assembled into unigenes, while most reads over 500 bp were assembled into unigenes, indicating that longer reads (>500 bp) are more likely to be assembled into unigenes.

**TABLE 1:** Summary of *A. germari* antennae transcriptome.

**Statistics project**	**Number**
Total raw reads	49,729,910
Total clean reads	47,631,128
Clean bases	7.14G
Q20 percentage	97.79%
Q30 percentage	94.26%
GC percentage	41.92%
Transcripts	115,952
Mean length of transcripts	647
N50 of transcripts	1007
Unigenes	62,299
Mean length of Unigenes	948
N50 of Unigenes	1295

**FIGURE 1:**
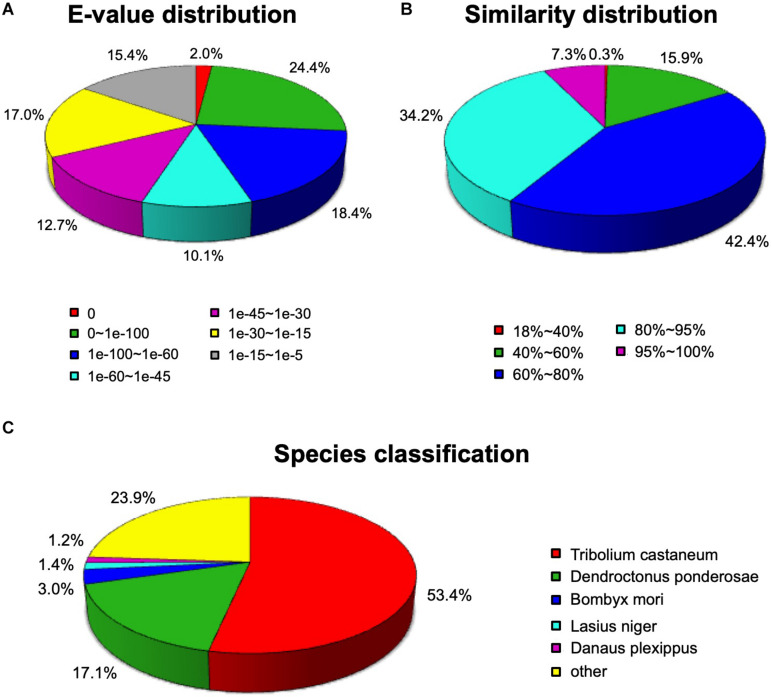
Homology analysis of *Apriona germari* unigenes. **(A)** E-value distribution. **(B)** Similarity distribution. **(C)** Species distribution. All unigenes that had BLASTX annotations within the NCBI nr database with a cutoff *E*-value of 10^–5^ were analyzed. The first hit of each sequence was used for analysis.

A total of 36,834 unigenes (59.12%) were compared to proteins in the NCBI Nr protein database using the BLASTX algorithm (*E*-value < 0.00001). As shown in Figure 1, among the annotated unigenes, approximately 44.8% unigenes showed a high homology (*E*-value < 1e-60) (Figure 1A). The identity comparison showed that 83.9% unigenes have more than 60% identity with other insects (Figure 1B). The top five species distributions were shown in Figure 1C. Approximately 76.1% unigenes were annotated to five top-hit insect species. *T. castaneum* was the first top-hit species with 53.4% annotated genes. The other top-hit species were *Dendroctonus ponderosae* (17.1%), *Bombyx mori* (3.0%), *Lasius niger* (1.4%), and *Danaus plexippus* (1.2%) (Figure 1C).

### Gene Ontology Annotation and KEGG Analysis

**FIGURE 2:**
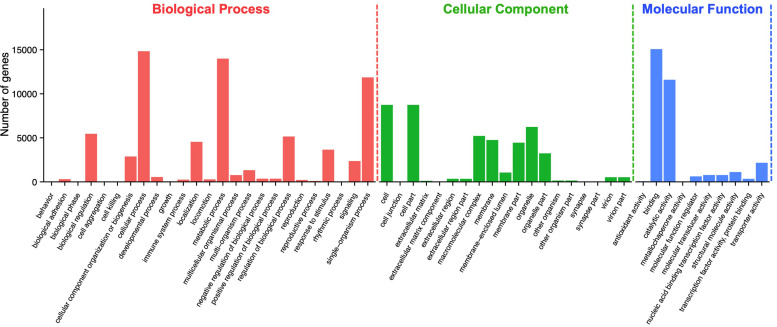
Gene ontology (GO) assignment of *Apriona germari* unigenes. The GO classification map was done by uploading the GO ID numbers of genes for their involvement in biological processes, cellular components, and molecular functions.

According to Gene Ontology (GO) analysis, a total of 27,260 unigenes were assigned to three GO functional categories: biological process, cellular component, and molecular function. Among them, 69,435 times are classified to the category of biological process, 44,677 times to cellular component, and 32,562 times to molecular function (Figure 2). A total of 54 categories were divided into subcategories: biological process (25 subcategories), cellular component (19 subcategories), and molecular function (10 subcategories). Among the 25 subcategories of biological process, the largest proportion of genes is involved in cellular process (14,836; 21.37%), followed by metabolic process (13,995; 20.16%) and single-organism process (11,864; 17.09%). As for the category of cellular component, cell (8,743; 19.57%) and cell part (8,743; 19.57%), followed by organelle (6,240; 13.97%), made up the majority of the proportion. For the category of molecular function, a significant proportion of the genes is assigned to binding (15,075; 46.3%) and catalytic activity (11,594; 35.61%), while no gene was assigned to antioxidant activity and metallochaperone activity (Supplementary Table S2).

**FIGURE 3:**
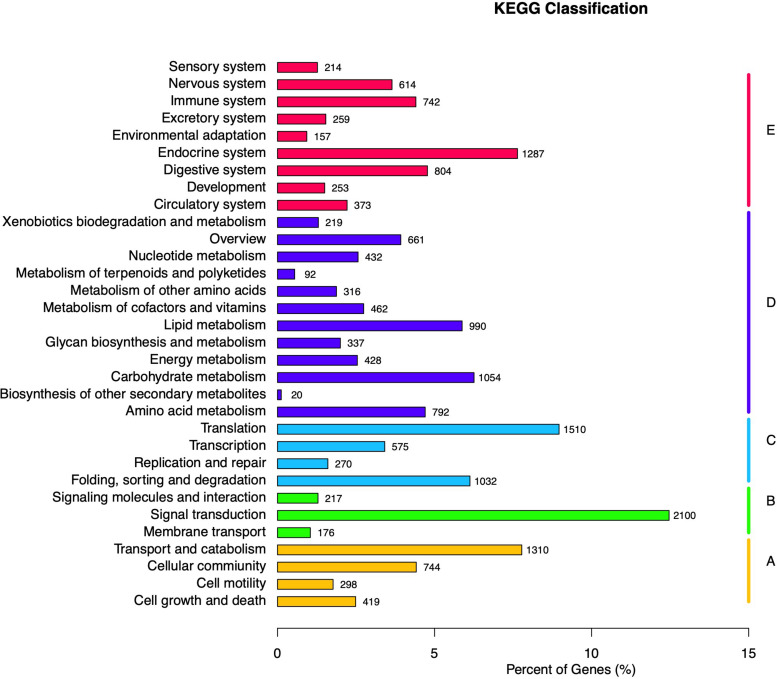
Kyoto Encyclopedia of Genes and Genomes (KEGG) classification of *Apriona germari* unigene. The *x*-axis indicates the percentage of annotated genes, and the y-axis indicates the KEGG categories. The capital letters against the colored bars indicate five main categories: **(A)** cellular processes, **(B)** environmental information processing, **(C)** genetic information processing, **(D)** metabolism, and **(E)** organism systems.

KEGG database was used to analyze the metabolic pathways of unigenes. A total of 16,846 unigenes were assigned to five specific KEGG pathways (Figures 3A–E): cellular processes (Figure 3A), environmental information processing (Figure 3B), genetic information processing (Figure 3C), metabolism (Figure 3D), and organismal systems (Figure 3E). Moreover, 32 KEGG pathways were further assigned to these five pathways, in which the largest number of unigenes (*n* = 2,100) was assigned to the pathway of signal transduction, followed by transduction (*n* = 1,510), transport and catabolism (*n* = 1,310), and endocrine system (*n* = 1,287).

### Identification of Putative Odorant Receptors

**FIGURE 4:**
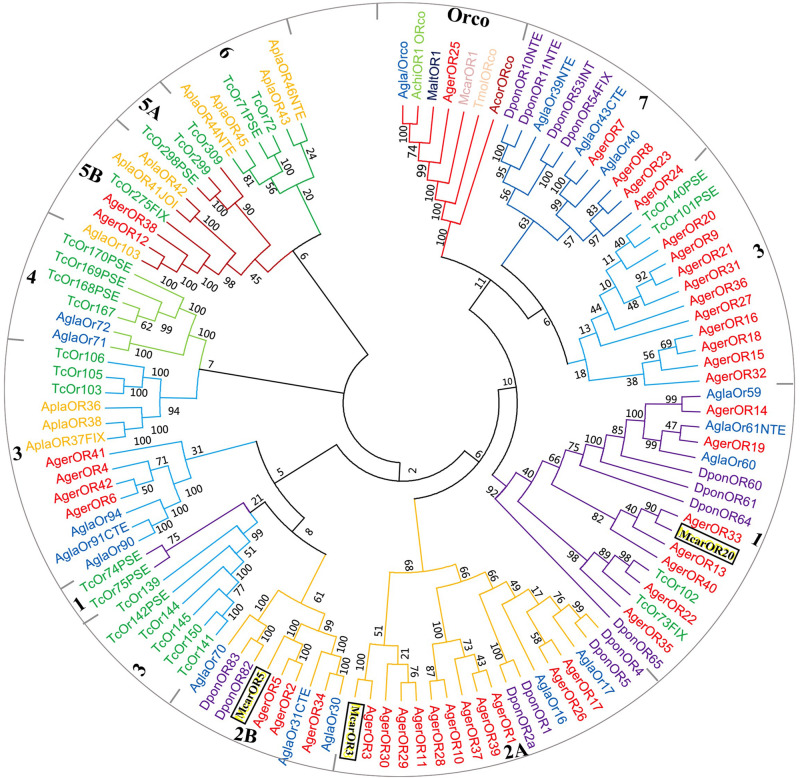
Molecular phylogeny comparing AgerORs with odorant receptors (ORs) from 10 insect species. A total of 42 ORs (AgerOR1–42) from *Apriona germari* (Ager) and ORs from *Anoplophora chinensis* (Achi), *Anoplophora glabripennis* (Agla), *Agrilus planipennis* (Apla), *Dendroctonus ponderosae* (Dpon), *Tribolium castaneum* (Tcas), *Anomala corpulenta* (Acor), *Monochamus alternates* (Malt), *Megacyllene caryae* (Mcar), and *Tenebrio molitor* (Tmol) were used to construct the phylogenetic tree (see section “Materials and Methods” for details of the phylogenetic analysis).

Based on the comparative analysis of the *A. germari* antennal transcriptome using Blastx databases, a total of 42 putative AgerORs genes were identified. As shown in Table 2, eight AgerORs (AgerOR1, 3, 8, 10, 25, 28, 37, and 41) were over 1,000 bp in length. However, only the AgerOR25 sequence had complete ORFs. Since AgerOR25 was identified with a high sequence homology with the conserved Orco gene family of other insect species, we therefore designated it as AgerOrco. Next, a phylogenetic tree was constructed to evaluate the relationships of AgerORs with other insects’ ORs (Figure 4). Based on the OR phylogenetic tree analysis, the published OR genes could be divided into multiple subgroups (numbered 1, 2A, 2B, 3, 4, 5A, 5B, 6, and 7). In the current study, except AgerOR25, which was designated as AgerOrco, the other 41 putative AgerORs were classified into five subgroups (group 1, 2A and 2B, 3, 5B, and 7). Seven AgerORs (AgerOR13, 14, 19, 22, 33, 35, and 40) were clustered in group 1, while 14 AgerORs (AgerOR1–3, 5, 10, 11, 17, 26, 28–30, 34, 37, and 39) were assigned to group 2A and 2B. Another 14 AgerORs (AgerOR4, 6, 9, 15, 16, 18, 20, 21, 27, 31, 32, 36, 41, and 42) belonged to group 3. AgerOR12 and AgerOR38 were clustered in group 5B. The other four AgerORs, including AgerOR7, 8, 23, and 24, were classed in group 7. In addition, seven AgerORs (AgerOR2, 3, 5, 13, 33, 34, and 40) were clustered with pheromone receptors from *M. caryae* (labeled in black square in Figure 4).

**TABLE 2:** Blastx match of *A. germari* OR genes.

**Number**	**ORF length(aa)**	**Complete ORF**	**FPKM**	**Best Blastx Match**				
				**Protein**	**Accession**	**Species**	***E*-value**	**Identity (%)**
AgerOR1	375	No	3.05	Odorant receptor 4-like	XP_018568462.1	*A. glabripennis*	3E-142	64
AgerOR2	102	No	2.15	Odorant receptor 4-like	XP_018577142.1	*A. glabripennis*	2.00E-73	80
AgerOR3	391	No	1.37	Odorant receptor Or1-like	XP_018564808.1	*A. glabripennis*	0	83
AgerOR4	124	No	2.42	Odorant receptor 94a-like	XP_018560823.1	*A. glabripennis*	2.00E-46	56
AgerOR5	200	No	7.93	Odorant receptor 4-like	XP_018577142.1	*A. glabripennis*	6.00E-134	91
AgerOR6	138	No	1.37	Odorant receptor 94a-like	XP_018560823.1	*A. glabripennis*	5.00E-54	56
AgerOR7	85	No	9	Odorant receptor Or2-like	XP_018562805.1	*A. glabripennis*	2.00E-28	65
AgerOR8	391	No	18.16	Odorant receptor Or2-like	XP_018569507.1	*A. glabripennis*	3.00E-88	42
AgerOR9	36	No	3.06	Putative odorant receptor 92a	XP_018572304.1	*A. glabripennis*	2.00E-64	61
AgerOR10	366	No	8.92	Odorant receptor 94a-like isoform X1	XP_018569520.1	*A. glabripennis*	9.00E-116	47
AgerOR11	205	No	3.01	Odorant receptor Or1-like	XP_018566565.1	*A. glabripennis*	2.00E-127	84
AgerOR12	188	No	3.02	Putative odorant receptor 71a	XP_018575789.2	*A. glabripennis*	4.00E-112	77
AgerOR13	248	No	3.84	Odorant receptor Or2-like	XP_018567969.1	*A. glabripennis*	4.00E-123	70
AgerOR14	117	No	3.03	Odorant receptor Or2-like	XP_018579015.1	*A. glabripennis*	1.00E-42	67
AgerOR15	133	No	1.48	Odorant receptor 43a-like	XP_018573343.1	*A. glabripennis*	2.00E-23	48
AgerOR16	45	No	1.82	Odorant receptor 49b-like	XP_018578867.1	*A. glabripennis*	9.00E-32	76
AgerOR17	97	No	5.27	Odorant receptor 85b-like	XP_018564120.1	*A. glabripennis*	1.00E-57	85
AgerOR18	144	No	1.23	Odorant receptor 85b-like	XP_018564120.1	*A. glabripennis*	1.00E-68	70
AgerOR19	115	No	8.67	Odorant receptor Or2-like	XP_018579015.1	*A. glabripennis*	1.00E-70	78
AgerOR20	49	No	2.81	Odorant receptor OR32	ALR72575.1	*C. bowringi*	2.00E-10	39
AgerOR21	220	No	0.22	Odorant receptor Or1-like	XP_018566505.1	*A. glabripennis*	4.00E-54	70
AgerOR22	153	No	1.87	Odorant receptor 49b-like	XP_018570955.1	*A. glabripennis*	4.00E-89	88
AgerOR23	62	No	4.05	Odorant receptor	AUF73032.1	*A. chinensis*	1.00E-27	55
AgerOR24	132	No	2.77	Odorant receptor	AUF73026.1	*A. chinensis*	3.00E-71	78
AgerOR25 Orco	480	Yes	23.61	Odorant receptor coreceptor	XP_018568191.1	*A. glabripennis*	0	95
AgerOR26	294	No	3.12	Odorant receptor Or2-like	XP_023311850.1	*A. glabripennis*	0.00E+00	81
AgerOR27	135	No	1.95	Odorant receptor 94a-like	XP_018560823.1	*A. glabripennis*	1.00E-31	47
AgerOR28	363	No	2.27	Odorant receptor 94a-like isoform X1	XP_018569520.1	*A. glabripennis*	2.00E-103	42
AgerOR29	268	No	2.16	Odorant receptor 1	APC94305.1	*P. aenescens*	9.00E-54	44
AgerOR30	194	No	4.19	Putative odorant receptor 71a	XP_018560835.1	*A. glabripennis*	3.00E-120	67
AgerOR31	220	No	1.7	Odorant receptor Or2-like	XP_018576526.1	*A. glabripennis*	1.00E-07	23
AgerOR32	84	No	4.73	Odorant receptor 43a-like	XP_018573343.1	*A. glabripennis*	1.00E-30	58
AgerOR33	101	No	2.97	Putative odorant receptor 92a	XP_023310462.1	*A. glabripennis*	6.00E-65	69
AgerOR34	91	No	0.74	Odorant receptor 59a-like	XP_018575063.1	*A. glabripennis*	7.00E-54	82
AgerOR35	126	No	2.37	Odorant receptor 67c-like	XP_018571501.1	*A. glabripennis*	1.00E-49	74
AgerOR36	218	No	1.35	Odorant receptor 4-like	XP_018577142.1	*A. glabripennis*	9.00E-100	64
AgerOR37	350	No	5.81	Odorant receptor	AUF73024.1	*A. chinensis*	6.00E-89	45
AgerOR38	186	No	1.47	Odorant receptor Or1-like	XP_018575790.1	*A. glabripennis*	5.00E-87	67
AgerOR39	190	No	2.38	Odorant receptor 94a-like isoform X1	XP_018569520.1	*A. glabripennis*	2.00E-109	78
AgerOR40	182	No	9.04	Odorant receptor 49b-like	XP_018578867.1	*A. glabripennis*	6.00E-66	56
AgerOR41	377	No	6.72	Odorant receptor 67c-like	XP_018561909.1	*A. glabripennis*	2.00E-120	60
AgerOR42	181	No	3.36	Odorant receptor 94a-like	XP_018560823.1	*A. glabripennis*	3.00E-94	57

### Tissue- and Sex-Specific Expressions of Odorant Receptors

**FIGURE 5:**
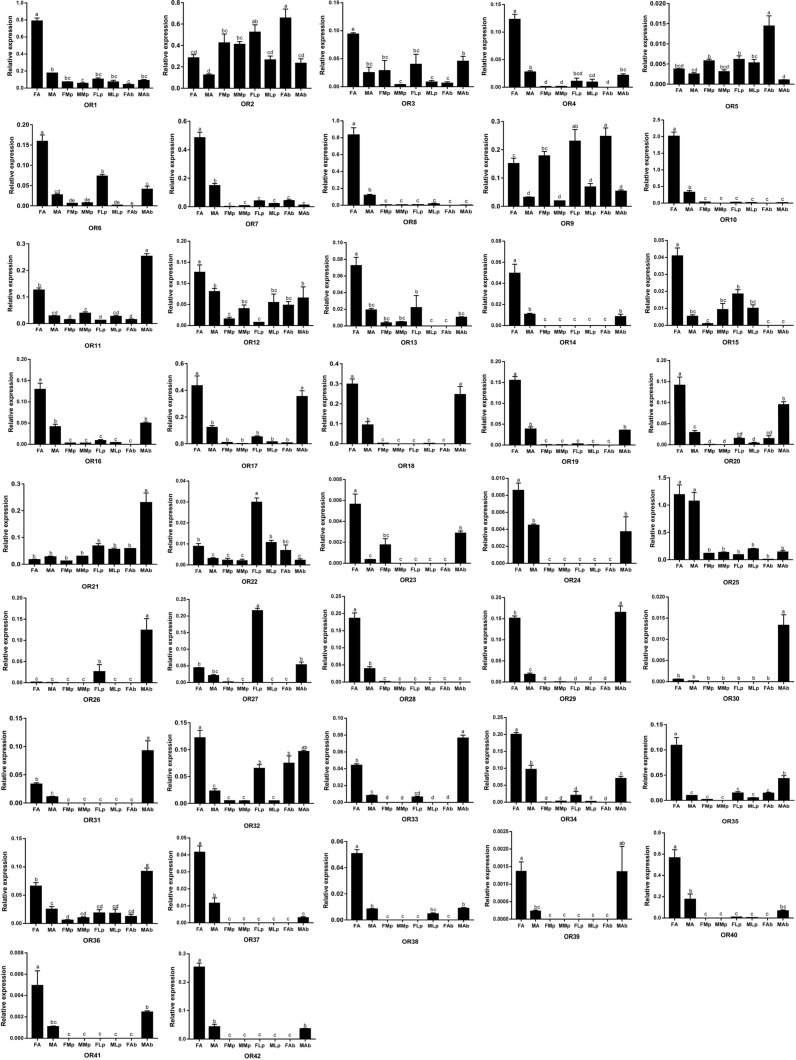
Relative mRNA expression of AgerORs in *Apriona germari* tissues. The relative mRNA levels were normalized to those of the actin gene and analyzed using the Q-gene method. All values are shown as mean ± SEM normalized. The data were analyzed by least significant difference test after one-way analysis of variance. Different letters (a–e) indicate significant differences between means (*P* < 0.05). FA, female antennae; MA, male antennae; FMp, female maxillary palps; MMp, male maxillary palps; FLp, female labial palps; MLp, male labial palps; FAb, female abdominal end; MAb, male abdominal end.

We next examined the expression of OR genes in adult female and male antennae, maxillary palps, labial palps, and the end part of the abdomen (abdominal end) by qRT-PCR with primers specific for each of the 42 AgerOR genes. AgerOR1, 2, 9, 11, 12, 21, 22, 32, and 36 were found to be ubiquitously expressed in chemosensory organs. In addition, the amplification products of 10, two, two, and two AgerOR genes were identified in the antennae, the maxillary palps, the labial palps, and the abdominal end, respectively (Figure 5 and Supplementary Figure S2).

Of the 40 AgerORs expressed in the antennae, 27 AgerORs (AgerOR1, 3, 4, 6–8, 10, 12–20, 23, 24, 28, 29, 34, 35, 37, 38, 40, and 42) were female-biased and two AgerORs (AgerOR21 and 25) were, of the same level, expressed in both female and male (Figure 5 and Supplementary Figure S2A). Among the 14 AgerOR genes expressed in the maxillary palps, AgerOR3, AgerOR5, and AgerOR9 were female-biased, while AgerOR11 were expressed at a significantly higher level in the male maxillary palps (Figure 5 and Supplementary Figure S2B). Among the 17 AgerOR genes expressed in the labial palps, AgerOR2, 9, and 27 were highly expressed in the female labial palps (Figure 5 and Supplementary Figure S2C). Finally, among the 38 abdominal end AgerORs analyzed, three AgerORs (AgerOR2, 5, and 9) were highly expressed in the female abdominal end, 13 AgerORs (AgerOR11, 17, 18, 20, 21, 25, 29, 31–34, 36, and 40) showed a high expression in the male end-body (Figure 5 and Supplementary Figure S2D).

### Identification of Putative Ionotropic Receptors and Their Expression Pattern

**FIGURE 6:**
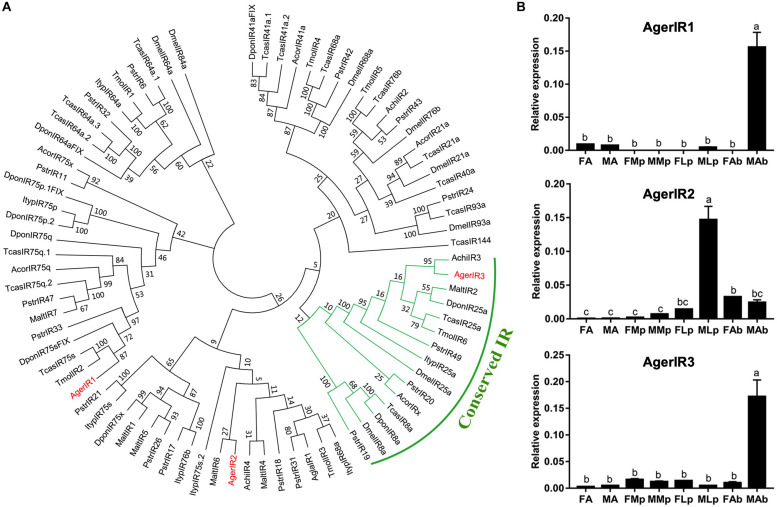
Phylogenetic tree and relative mRNA expression of AgerIRs. **(A)** A total of 42 ORs (AgerOR1–42) from *Apriona germari* (Ager) and ORs from *Anoplophora chinensis* (Achi), *Anoplophora glabripennis* (Agla), *Dendroctonus ponderosae* (Dpon), *Drosophila melanogaster* (Dmel), *Phyllotreta striolata* (Pstr), *Anomala corpulenta* (Acor), *Monochamus alternates* (Malt), *Megacyllene caryae* (Mcar), *Tenebrio molitor* (Tmol), and *Tribolium castaneum* (Tcas) were used to construct the phylogenetic tree. **(B)** Relative mRNA expression of AgerIRs. The relative mRNA levels were normalized to those of the *actin* gene and analyzed using the Q-gene method. All values are shown as mean ± SEM normalized. The data were analyzed by least significant difference test after one-way analysis of variance. Different letters (a–c) indicate significant differences between means (*P* < 0.05). FA, female antennae; MA, male antennae; FMp, female maxillary palps; MMp, male maxillary palps; FLp, female labial palps; MLp, male labial palps; FAb, female abdominal end; MAb, male abdominal end.

In the current study, three putative AgerIRs were also identified in the combined antennal transcriptome (Table 3). According to the phylogenetic analysis of IRs from one Diptera *D. melanogaster* and eight species of Coleopterans (Figure 6A), AgerIR3 was clustered with TcasIR25a at high percent identity, suggesting that it belongs to the IR25a coreceptor subfamily.

**TABLE 3:** Blastx match of *A. germari* IR genes.

**Number**	**ORF Length(aa)**	**Complete ORF**	**FPKM**	**Best Blastx Match**				
				**Protein**	**Accession**	**Species**	***E*-value**	**Identity (%)**
AgerIR1	491	No	3.66	Ionotropic receptor IR2	ALR72541.1	*C. bowringi*	0	68
AgerIR2	264	No	5.65	Glutamate receptor ionotropic, delta-1	XP_018568700.1	*A. glabripennis*	7.00E-135	77
AgerIR3	944	No	1.29	Ionotropic receptor 25a	XP_018574744.1	*A. glabripennis*	0	91

To further investigate the expression of these three IR genes, qRT-PCR experiments were also performed using total RNA prepared from male antennae, maxillary palps, labial palps, and the end part of the abdomen (abdominal end) taken from both female and male *A. germari* adults. As shown in Figure 6B, AgerIR1 and AgerIR3 were found in the male end-body, with a significantly higher expression than in any other tissues. Another AgerIR gene, AgerIR2, showed high expression levels in the male labial palps (Figure 6B and Supplementary Figure S3).

## Discussion

Coleoptera is the first order of Insecta and one of the most important orders in forestry, and the longhorn beetle is one of the larger groups of Coleoptera, with 45,000 species in the world and more than 3,100 species known in China (Zhang, 2011). To date, however, olfactory genes have been studied in only 20 species and limited in three species of Cerambycidae (*Monochamus alternatus*, *Batocera horsfieldi*, and *A. glabripennis*) (Li et al., 2014; Gao and Wang, 2015; Hu et al., 2016). *A. germari* (Hope) (Coleoptera: Cerambycidae) is one of the most serious insect pests that cause damage to economic trees and landscaping trees, resulting in massive environmental damages and economic losses. To reveal the olfactory receptor genes involved in this beetle, we conducted an RNA sequencing analysis of the antennae of adult *A. germari*.

In the transcriptome sets, we obtained 62,299 unigenes from 115,952 transcripts, with a mean length of 948 bp, and 67.07% of these unigenes were longer than 500 bp. These results indicate the high quality and depth of the transcriptome sequences. The BLASTX homology analysis showed that *A. germari* antennal transcriptome sequences best match with *T. castaneum* (53.4%) and *D. ponderosae* (17.1%), presumably because many homologous genes, including olfactory genes, were present in *T. castaneum*, *D. ponderosae*, and *A. germari*. The above unigenes may play a significant part in antennal chemosensory processes, such as transporter activity and odorant binding.

The 42 AgerORs identified in *A. germari* are similar to the 43 OR genes in *A. corpulenta* (Li et al., 2015) and *I. typographus* (Andersson et al., 2013), fewer than the 57 ORs reported in *M. caryae* (Mitchell et al., 2012) or the 73 transcripts encoding ORs in the antennal transcriptomes of *P. striolata* (Wu et al., 2016) and the 53 ORs in *A. chinensis* but more than the nine ORs identified in *M. alternatus* (Wang et al., 2014), six ORs in *Rhyzopertha dominica* (Diakite et al., 2016), or 37 ORs in *A. glabripennis* (Hu et al., 2016) belonging to seven known coleopteran specific subgroups. There were seven coleopteran specific subgroups reported in previous studies (Engsontia et al., 2008; Andersson et al., 2013, 2019). In the phylogenetic tree of ORs, 42 putative AgerORs sequences were spread into five subgroups (Figure 4). Interestingly, five AgerORs (AgerOR3, 5, 13, 33, and 40) were clustered with three pheromone receptors (PRs: McarOR3, McarOR5, and McarOR20), which are functionally characterized receptors from the cerambycid beetle *M. caryae* (Mitchell et al., 2012). Among the five AgerORs, AgerOR3 was orthologous to McarOR3, a receptor sensitive to the cerambycid pheromone (*S*)-2-methyl-1-butanol. Moreover, AgerOR5 was gathered with McarOR5, which is sensitive to 2-phenylethanol. In addition, AgerOR13, 33, and 40 were clustered with McarOR20, which was identified as a receptor of (2S, 3R)-2,3-hexanediol and 3-hydroxyhexan-2-one (Mitchell et al., 2012). Since they have high sequence similarities with the three PRs (McarOR3, McarOR5, and McarOR20) (Supplementary Figures S4–S6), these AgerORs may function to detect the abovementioned pheromones or other behaviorally active compounds.

Orco is the conserved subunit of ORs in insects and has been suggested as an attractive target for the manipulation of insect pest control programs (Kepchia et al., 2019). AgerOR25 could be the Orco in *A. germari* due to its specific Orco subgroup. Consistent with our previous report (Sun et al., 2018), the potential Orco (AgerOR25) showed a high sequence identity with MaltOR1 (Wang et al., 2014), McarOR1 (Mitchell et al., 2012), AglaOR1 (Mitchell et al., 2017), and AchiOR1 (Sun et al., 2018) (Supplementary Figure S7), indicating the conserved attribute of the Orco gene. Since the Orco plays a critical role in odorant detection, to disrupt this gene expression could be a potential utilization for control strategies. Orco silencing through RNA interference may hinder their ability to use olfactory cues to locate host plants and/or mates and to reduce pest populations. Overall, the highly conserved Orco genes of several Cerambycidae species could be anticipated as a potential interfering target for manipulation of the control programs.

Odor receptors are generally expressed in the antennae, but the specific function of odor receptors is different. In a recent study, 45 ORs were identified from the transcriptome of *A. chinensis* antennae. The qRT-PCR analysis showed that 41 ORs were highly expressed in the antennae; however, one OR was found to be highly expressed in the maxillary palp and the other three ORs were found to be highly expressed in the male body (Sun et al., 2018). Among the transcriptome of *Adelphocoris lineolatus* antennae with 57 randomly selected ORs, 26 ORs were found to be specifically expressed in male antennae, 16 ORs were found to be specifically expressed in female antennae, and the other ORs showed complex expression patterns, such as high expression in the body except the head or equal expression in male and female antennae (Xiao et al., 2017). In the current study, 27 AgerORs showed an antenna-specific expression profile. Those with female-biased expression may play a vital role in some female-specific behaviors such as oviposition site seeking. Notably, AgerOR3, 5, 13, 33, and 40 showed a clear female-biased expression profile (Figure 5). These five AgerORs were clustered with the pheromone receptors of *M. caryae* on the phylogenetic tree and have relatively similar amino acid sequences (Figure 4 and Supplementary Figures S4, S6). These findings allowed us to speculate that these receptors could be a potential pheromone receptor for sensing a male-produced sex pheromone in *A. germari*. In addition, we found that some AgerORs were highly expressed in the maxillary or the labial palp, suggesting that they may be involved in host selection for both sexes and oviposition site selection for females. We also found some AgerORs to be highly expressed in end-body tissues, which is consistent with what has been reported in other insects (Li et al., 2015; Zhang et al., 2016). In particular, 13 AgerORs showed a high expression in male end-body. Previous studies showed that some species of coleopteran, including *Callosobruchus chinensis* (Bruchidae), *Aleochara curtula* (Staphylinidae), and *Adalia bipunctata* (Coccinellidae), utilize cuticular hydrocarbons as contact sex pheromones (Tanaka et al., 1981; Peschke and Metzler, 1987; Hemptinne et al., 1998). We speculated that these AgerORs may play an important role related to function as the contact sex pheromone of *A. germari*.

IRs is a conserved family of synaptic ligand-gated ion channels, and its function has been reported to be involved not only in olfaction and gustation but also in thermosensation and hygrosensation (Missbach et al., 2014; Rimal and Lee, 2018). Three AgerIRs (AgerIR1–3) were identified in this study. The number of IRs of *A. germari* was less than that of *D. ponderosae* (*n* = 15) or *P. striata* (*n* = 49) (Wu et al., 2016) but relatively similar to *A. chinensis* (*n* = 4) (Sun et al., 2018), *A. glabripennis* (*n* = 4) (Hu et al., 2016), *A. corpulenta* (*n* = 5), and *M. alternates* (*n* = 7) (Wang et al., 2014). In the IR phylogenetic tree, IR25a and IR8a formed a conserved IR ortholog; *AgerIR3* was in the conserved IR orthologs, which indicates that AgerIR3 is the conserved IR of *A. germari*, and AgerIR2 clustered with IR76b clade. In *D. melanogaster*, IRs were found to be expressed in the olfactory and the gustatory organs with the detection of acids, amines, and aldehydes (Benton et al., 2009; Abuin et al., 2011; Depetris-Chauvin et al., 2015; Ganguly et al., 2017). In *A. glabripennis*, a previous study suggested that the maxillary and the labial palps of the male beetle play an important role in detecting this beetles’ female-deposited trail-sex pheromone blend (Graves et al., 2016). Based on the results that AgerIR2 was highly expressed in the male labial palps (Figure 6B and Supplementary Figure S3), we speculate that this receptor may function to detect the female-deposited trail-sex pheromone blend of *A. germari*. In addition, AgerIR1 and AgerIR3 were found to be expressed in the end-body, indicating that IRs are expressed mainly, but not limited, in the olfactory and the gustatory organs. In the current study, however, the IR8a clade in *A. germari* was missing, which may be attributed to the sequencing depth and coverage. Further studies by adopting next-generation sequencing technology would be needed to clarify the question.

In summary, we firstly conducted an RNA sequencing analysis of the antennae of adult *A. germari*. Forty-five putative receptor genes were identified from the olfactory receptor gene families, including 42 AgerORs and three AgerIRs, through bioinformatic analysis. The qRT-PCR demonstrated that most olfactory receptors were prominently expressed in the antennae, especially in female antennae, suggesting that they were involved in female-specific behaviors. The functions of these receptors are unknown but can be inferred from the receptors of other longhorns. AgerOR3, 5, 13, 33, and 40 were clustered with the pheromone receptors of *M. caryae*, implying that they may be sensitive to structurally related chemicals that are pheromones of *M. caryae*. AgerIR2 was highly expressed in the male labial palps, suggesting that it may function to detect the female-deposited trail-sex pheromone blend of *A. germari*. In addition, four known Orcos of currently reported cerambycidae species and a new Orco (AgerOR25) of *A. germari* identified in this study were highly conservative, which could be anticipated as a potential interfering target for the manipulation of control programs. Overall, our findings provide a theoretical basis for subsequent studies on the olfactory mechanisms of *A. germari* and offer some new insights into the functions and the evolutionary characteristics of ORs and IRs in Coleoptera insects.

## Data Availability Statement

The datasets generated for this study can be found in NCBI Genbank. The Genbank accession numbers were assigned as MT598219 to MT598257 for ORs and MT591677 to MT591679 for IRs.

## Author Contributions

L-WZ, J-LQ, and Z-QL conceived and designed the experiments. J-LQ, G-CL, JY, and BC performed the experiments. J-LQ, L-WZ, D-ZM, BC, and LS analyzed the data. J-LQ, L-WZ, D-ZM, and Z-QL wrote the manuscript. All the authors contributed to the article and approved the submitted version.

## Conflict of Interest

The authors declare that the research was conducted in the absence of any commercial or financial relationships that could be construed as a potential conflict of interest.

## Footnotes

1 https://www.ncbi.nlm.nih.gov/orffinder/

2 http://www.cbs.dtu.dk/services/TMHMM/

## Supplementary Material

FIGURE S1 Sequence length distribution of transcripts and unigenes assembled from Illumina reads for the mantle transcriptome of *Apriona germari*. The *x*-axis indicates the length interval of transcripts and unigenes, and the *y*-axis indicates the number of transcripts and unigenes for each size.

FIGURE S2 Relative mRNA expression of AgerORs in *Apriona germari* tissues. The relative mRNA levels were normalized to those of the actin gene and analyzed using the Q-gene method. All values are shown as mean ± SEM normalized. The data were analyzed by least significant difference test after one-way analysis of variance.

FIGURE S3 Relative mRNA expression of AgerIRs in *Apriona germari* tissues. The relative mRNA levels were normalized to those of the actin gene and analyzed using the Q-gene method. All values are shown as mean ± SEM normalized. The data were analyzed by least significant difference test after one-way analysis of variance.

FIGURE S4 Multiple sequence alignment of *Megacyllene caryae* and *Apriona germari* ORs. The multiple alignment and the homology of each OR were calculated using ClustalW2 (http://www.ebi.ac.uk/Tools/msa/clustalw2/).

FIGURE S5 Sequence alignment of McarOR3 and AgerOR3. The multiple alignment and the homology of each OR were calculated using ClustalW2 (http://www.ebi.ac.uk/Tools/msa/clustalw2/).

FIGURE S6 Sequence alignment of McarOR5 and AgerOR5. The multiple alignment and the homology of each OR were calculated using ClustalW2 (http://www.ebi.ac.uk/Tools/msa/clustalw2/).

FIGURE S7 Alignment of four known Orcos and a new Orco (AgerOR25) of *Apriona germari* identified in this study. Malter, *M. alternates*; Mcar, *M. caryae*; Agla, *A. glabripennis*; Achi, *A. chinensis*; Ager, *A. germari.* The multiple alignment and the homology of each Orco were calculated using ClustalW2 (http://www.ebi.ac.uk/Tools/msa/clustalw2/).

TABLE S1 Primers of *Apriona germari* olfactory receptor genes used for quantitative real-time polymerase chain reaction.

TABLE S2 Gene Ontology classification count of *Apriona germari* antennae transcriptome.
