# Supplementary figures and images for: Identification and Expression Profile of Olfactory Receptor Genes Based on Apriona germari (Hope) Antennal Transcriptome
Source: Front Physiol. 2020 Jul 22;11:807. doi: 10.3389/fphys.2020.00807 (PMC7387575; doi:10.3389/fphys.2020.00807)

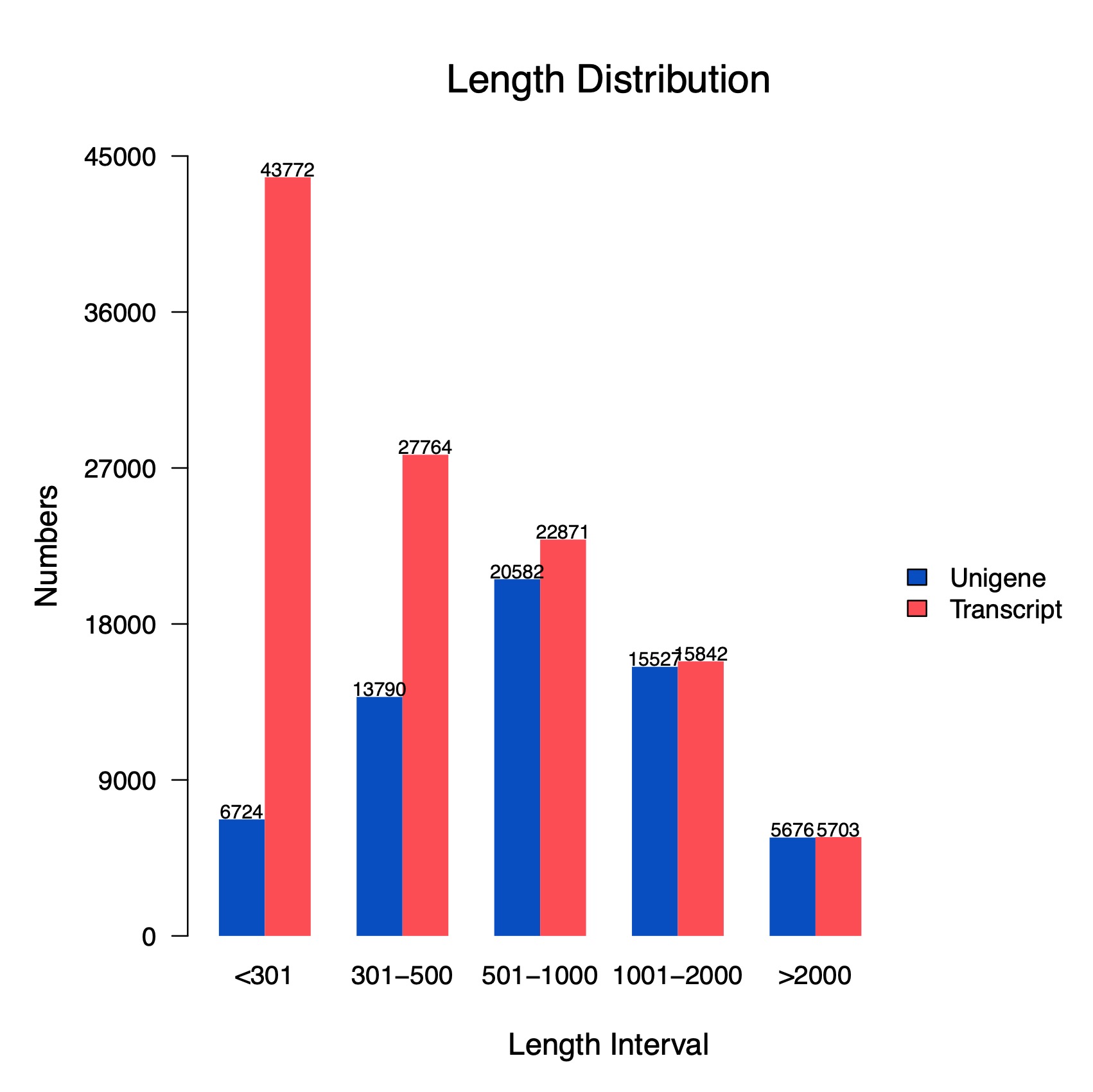

Supplement: FIGURE S1 — Sequence length distribution of transcripts and unigenes assembled from Illumina reads for the mantle transcriptome of Apriona germari. The x-axis indicates the length interval of transcripts and unigenes, and the y-axis indicates the number of transcripts and unigenes for each size. [file Image_1.JPEG]

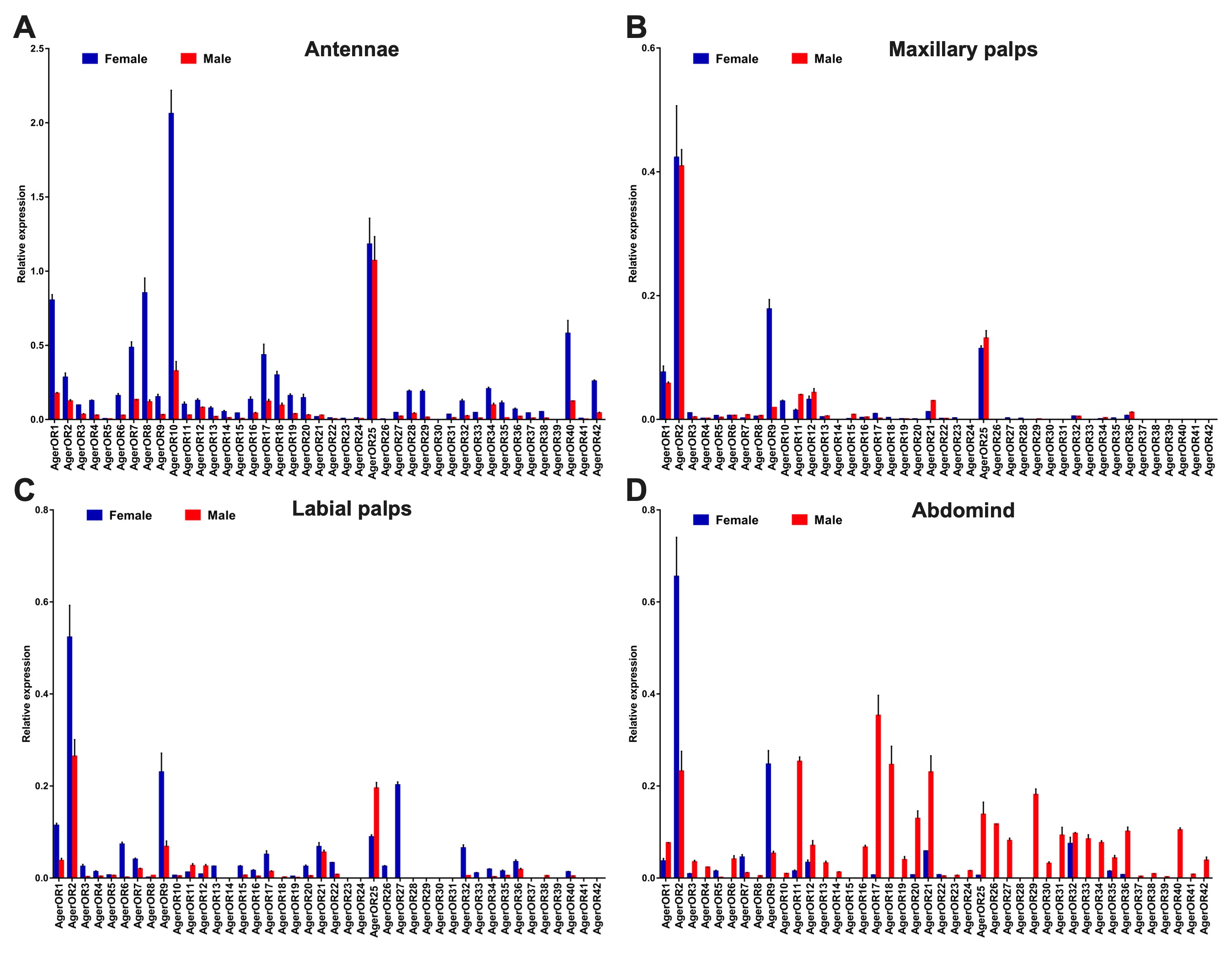

Supplement: FIGURE S2 — Relative mRNA expression of AgerORs in Apriona germari tissues. The relative mRNA levels were normalized to those of the actin gene and analyzed using the Q-gene method. All values are shown as mean ± SEM normalized. The data were analyzed by least significant difference test after one-way analysis of variance. [file Image_2.JPEG]

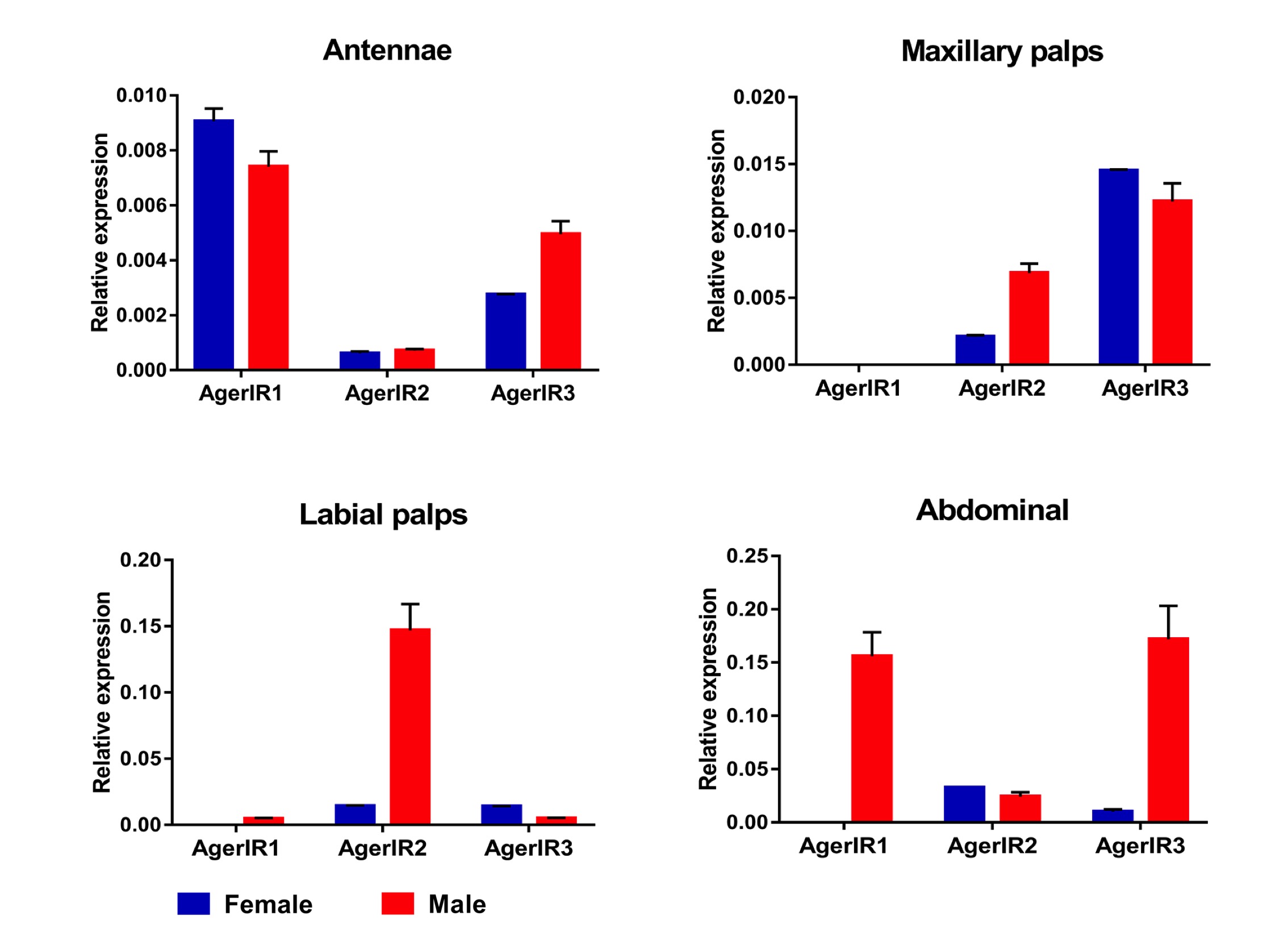

Supplement: FIGURE S3 — Relative mRNA expression of AgerIRs in Apriona germari tissues. The relative mRNA levels were normalized to those of the actin gene and analyzed using the Q-gene method. All values are shown as mean ± SEM normalized. The data were analyzed by least significant difference test after one-way analysis of variance. [file Image_3.JPEG]

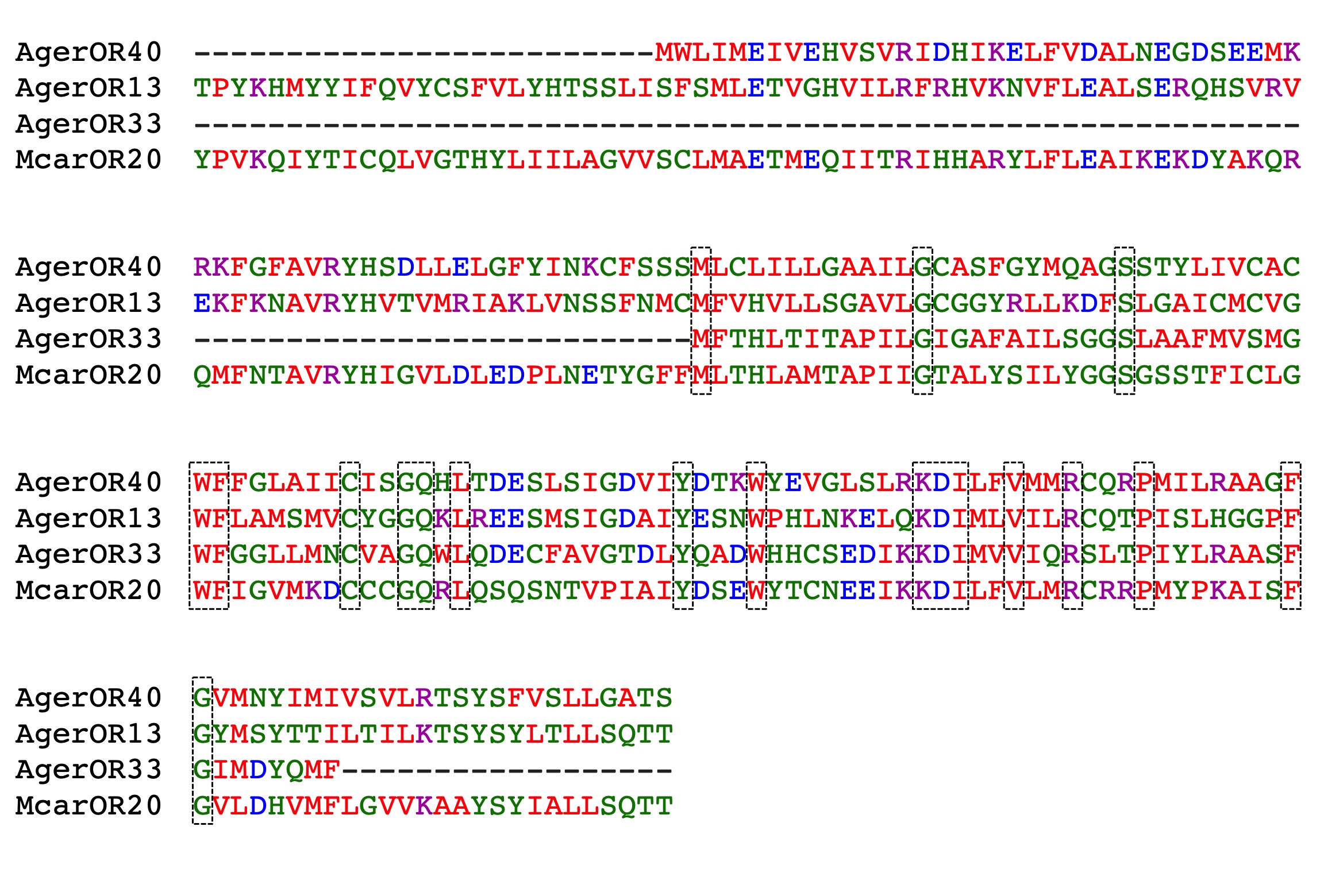

Supplement: FIGURE S4 — Multiple sequence alignment of Megacyllene caryae and Apriona germari ORs. The multiple alignment and the homology of each OR were calculated using ClustalW2 (http://www.ebi.ac.uk/Tools/msa/clustalw2/). [file Image_4.JPEG]

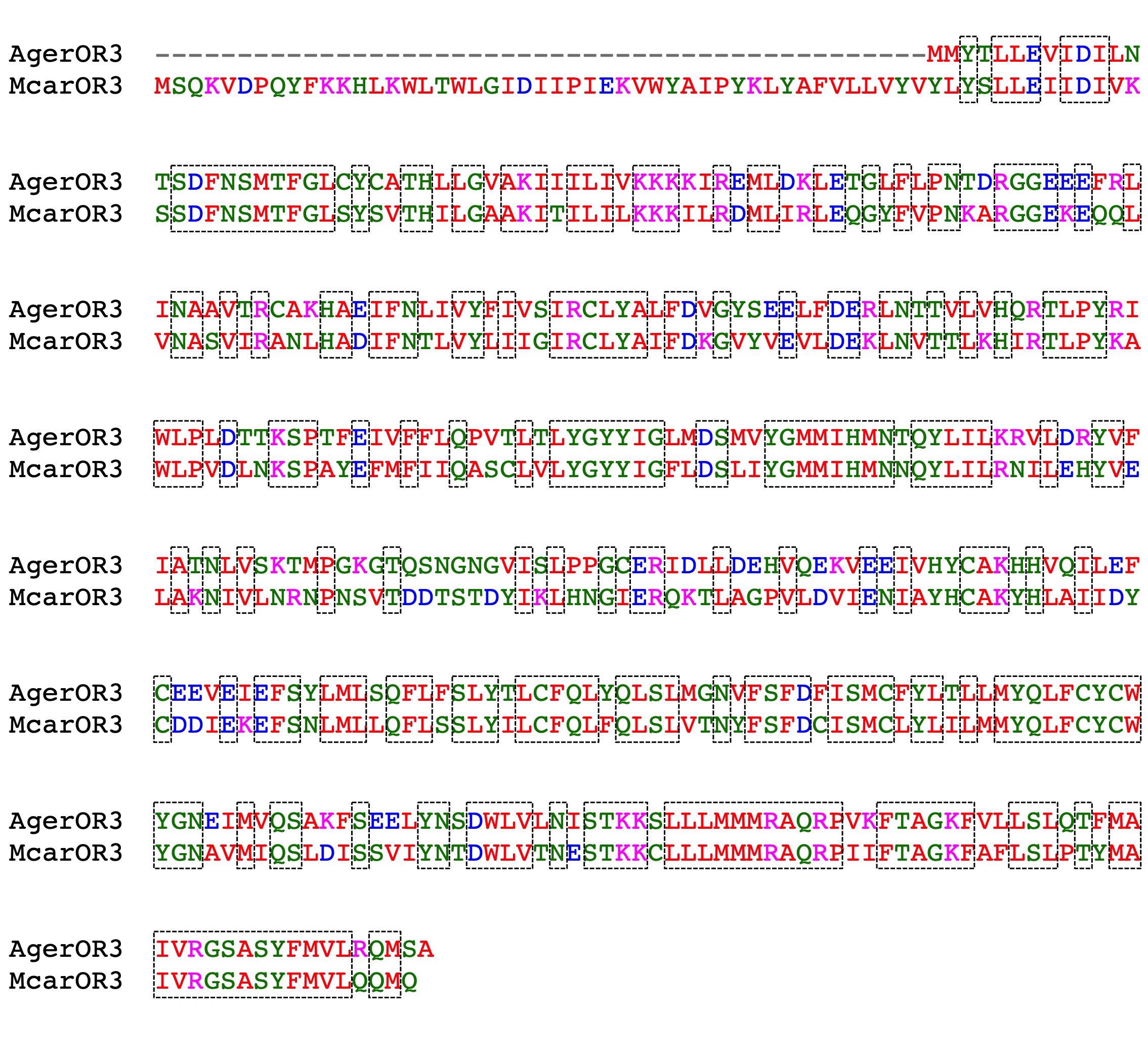

Supplement: FIGURE S5 — Sequence alignment of McarOR3 and AgerOR3. The multiple alignment and the homology of each OR were calculated using ClustalW2 (http://www.ebi.ac.uk/Tools/msa/clustalw2/). [file Image_5.JPEG]

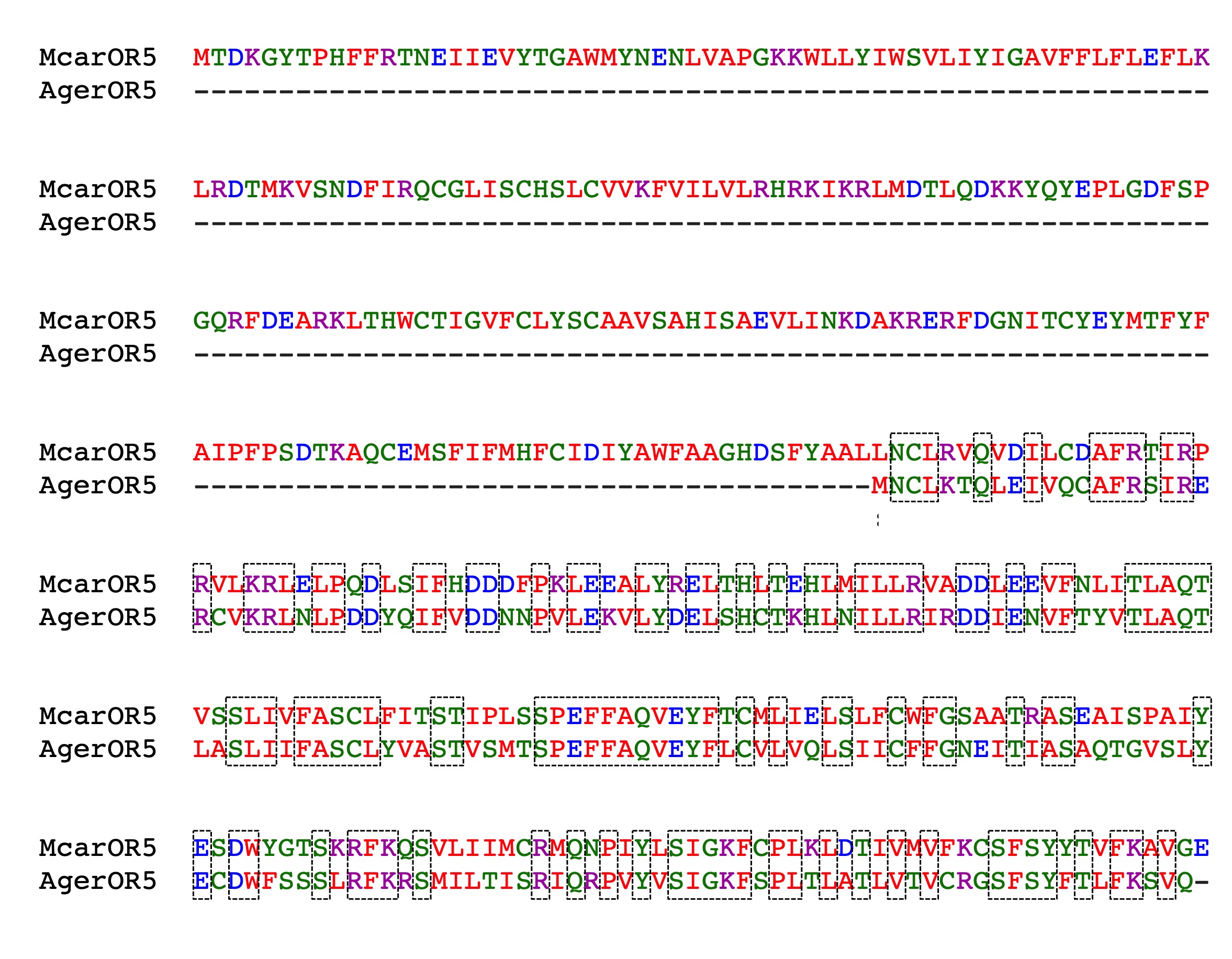

Supplement: FIGURE S6 — Sequence alignment of McarOR5 and AgerOR5. The multiple alignment and the homology of each OR were calculated using ClustalW2 (http://www.ebi.ac.uk/Tools/msa/clustalw2/). [file Image_6.JPEG]

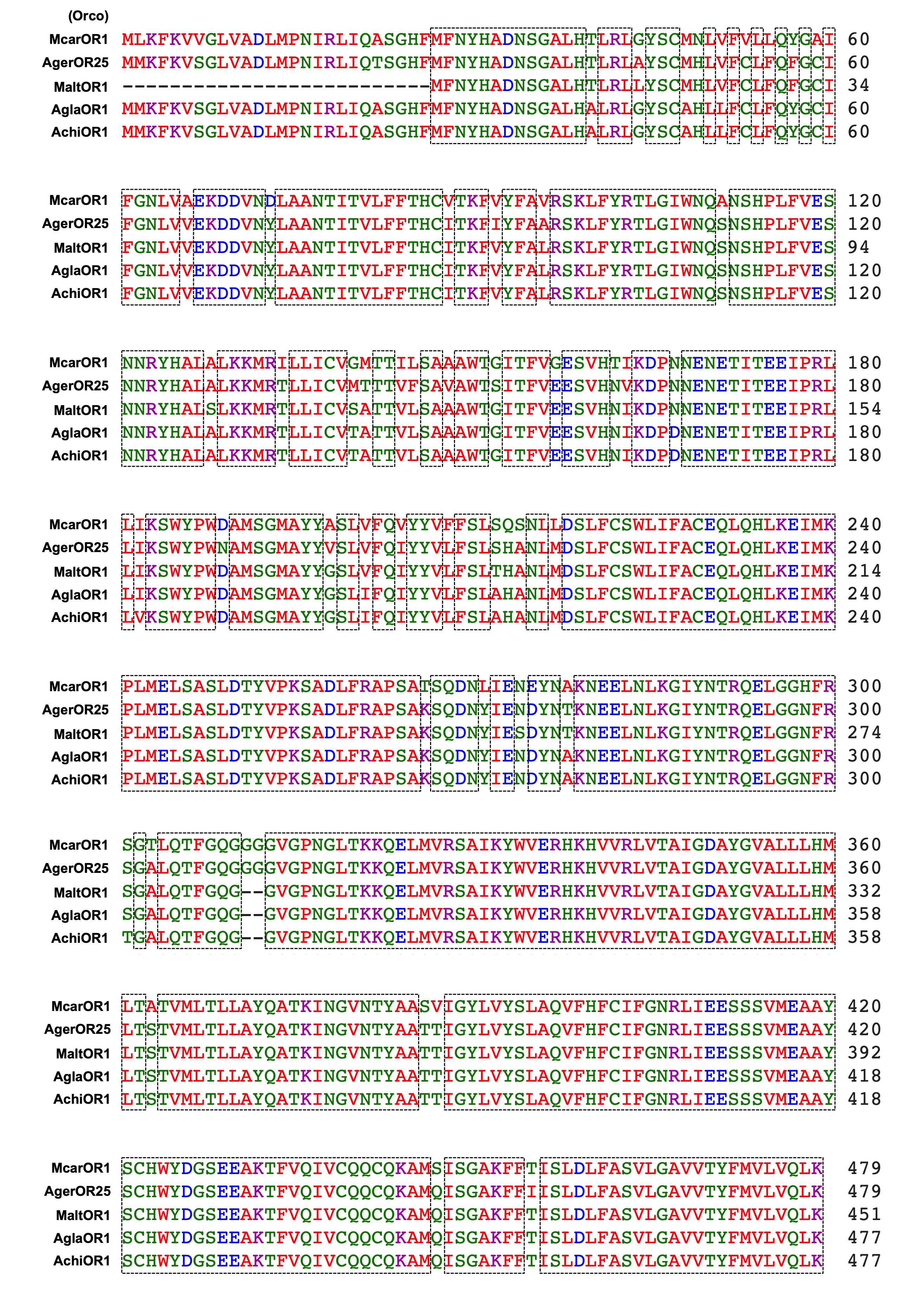

Supplement: FIGURE S7 — Alignment of four known Orcos and a new Orco (AgerOR25) of Apriona germari identified in this study. Malter, M. alternates; Mcar, M. caryae; Agla, A. glabripennis; Achi, A. chinensis; Ager, A. germari. The multiple alignment and the homology of each Orco were calculated using ClustalW2 (http://www.ebi.ac.uk/Tools/msa/clustalw2/). [file Image_7.JPEG]
