# Supplementary material for: Identification and Expression Profile of Olfactory Receptor Genes Based on Apriona germari (Hope) Antennal Transcriptome
Source: Front Physiol. 2020 Jul 22;11:807. doi: 10.3389/fphys.2020.00807 (PMC7387575; doi:10.3389/fphys.2020.00807)
Supplement: TABLE S1 — Primers of Apriona germari olfactory receptor genes used for quantitative real-time polymerase chain reaction. [file Table_1.docx]

TABLE S1 GO Classification count of *A. germari* antennae transcriptome

| GO Term (Lev2) | GO Term (Lev1) | Gene Number |
| --- | --- | --- |
| nucleic acid binding transcription factor activity | Molecular Function | 775 |
| metallochaperone activity | Molecular Function | 12 |
| molecular function regulator | Molecular Function | 624 |
| molecular transducer activity | Molecular Function | 781 |
| binding | Molecular Function | 15075 |
| transporter activity | Molecular Function | 2168 |
| antioxidant activity | Molecular Function | 71 |
| structural molecule activity | Molecular Function | 1118 |
| transcription factor activity, protein binding | Molecular Function | 344 |
| catalytic activity | Molecular Function | 11594 |
| biological adhesion | Biological Process | 310 |
| single-organism process | Biological Process | 11864 |
| rhythmic process | Biological Process | 16 |
| reproductive process | Biological Process | 107 |
| reproduction | Biological Process | 216 |
| cell aggregation | Biological Process | 3 |
| response to stimulus | Biological Process | 3655 |
| regulation of biological process | Biological Process | 5152 |
| cell killing | Biological Process | 27 |
| positive regulation of biological process | Biological Process | 361 |
| localization | Biological Process | 4545 |
| signaling | Biological Process | 2368 |
| negative regulation of biological process | Biological Process | 361 |
| growth | Biological Process | 50 |
| multicellular organismal process | Biological Process | 768 |
| metabolic process | Biological Process | 13995 |
| cellular component organization or biogenesis | Biological Process | 2882 |
| behavior | Biological Process | 21 |
| multi-organism process | Biological Process | 1329 |
| cellular process | Biological Process | 14836 |
| biological phase | Biological Process | 16 |
| developmental process | Biological Process | 553 |
| immune system process | Biological Process | 255 |
| locomotion | Biological Process | 285 |
| biological regulation | Biological Process | 5460 |
| synapse part | Cellular Component | 17 |
| membrane part | Cellular Component | 4451 |
| virion | Cellular Component | 528 |
| other organism | Cellular Component | 152 |
| macromolecular complex | Cellular Component | 5219 |
| extracellular matrix | Cellular Component | 117 |
| cell | Cellular Component | 8743 |
| other organism part | Cellular Component | 152 |
| membrane-enclosed lumen | Cellular Component | 1057 |
| extracellular region | Cellular Component | 349 |
| cell part | Cellular Component | 8743 |
| virion part | Cellular Component | 528 |
| extracellular matrix component | Cellular Component | 2 |
| cell junction | Cellular Component | 19 |
| membrane | Cellular Component | 4756 |
| organelle | Cellular Component | 6240 |
| synapse | Cellular Component | 17 |
| extracellular region part | Cellular Component | 347 |
| organelle part | Cellular Component | 3240 |
