# Supplementary material for: Identification and Expression Profile of Olfactory Receptor Genes Based on Apriona germari (Hope) Antennal Transcriptome
Source: Front Physiol. 2020 Jul 22;11:807. doi: 10.3389/fphys.2020.00807 (PMC7387575; doi:10.3389/fphys.2020.00807)
Supplement: TABLE S2 — Gene Ontology classification count of Apriona germari antennae transcriptome. [file Table_2.docx]

**TABLE S2** Primers of *A. germari* olfactory receptor genes used for RT-qPCR

| Gene name | Forward primer(5’-3’) | Reverse primer(5’-3’) |
| --- | --- | --- |
| ORs |  |  |
| AgerOR1 | AACCAGCCAGTCATAATG | GAATAAGCCGAACTCCATA |
| AgerOR2 | GCTCAGGTGGAATACTTC | CTAGCAACGGTAATCTCG |
| AgerOR3 | TACGGCAATGAGATTATGG | TTCTTGGTGGAGATGTTC |
| AgerOR4 | CGGAGACACGATATACTT | GCATAATCATAATCAGCAGAT |
| AgerOR5 | TTTCGCAGTATTAGAGAG | GAATCCGTAAGAGTATGT |
| AgerOR6 | CAGAACTTAACCGAATCA | CGTCCATTAGTAACTGAG |
| AgerOR7 | GATGATGATTATTATGCTGATGAG | ATGTTGCCTTGAGTATTCC |
| AgerOR8 | TTGTATTGGTATGCTCAT | CGTGCTTCTGTTATATTC |
| AgerOR9 | GAAGAAGAGAAGATCTAAGGTGAA | TTCCTCATCAGCAGGTTG |
| AgerOR10 | GCTATGGCAACTTGTATT | GGATGTATTGGCTCTATTC |
| AgerOR11 | ATTCATCGTCAGCGTTGT | TCCAGCAGCATACAGAAC |
| AgerOR12 | TGTATTAGATGCCGTGTA | GCTCTGAGTAGTGTAATG |
| AgerOR13 | GTATATTGTTCATTCGTGTT | CTTACTGAGTGTTGTCTT |
| AgerOR14 | AGAACAATGAAGGATATACT | TATGTAGATTGCTGAAGAA |
| AgerOR15 | AATTCTTGAGGCAATAGC | GGTCCAATGTTGATGATTA |
| AgerOR16 | AGGTGTGAAGCAAGCAAGT | TGGTAGTGGAATAACAGACAACAT |
| AgerOR17 | CTGGTATGAAGCAGAACA | GTATGAATAAGATAAGCGGATTA |
| AgerOR18 | ATGACAACCGTTATCTAC | CGCAAATACTACCCAATA |
| AgerOR19 | ATAACAGATGAGACATTG | GTGAGATAAGAGTAAGAC |
| AgerOR20 | CGAGGGTGCATTCCATTTATC | GCTGTTACTCTTTGGTTACTTTCT |
| AgerOR21 | CCTACTGGAACTGGATGA | CTACAACGTGGAAGTGAC |
| AgerOR22 | TGATGGATGACAGTTCTA | AAGTAGGAGTAAGCATTG |
| AgerOR23 | AACACCAGTATTCAGATTA | AACATGACATAAGTATAAGC |
| AgerOR24 | CTTGACTGTAGGACCATT | CGTAACTATGCTGAAGGA |
| AgerOR25 | ATTACGGTTCTATTCTTCAC | CCTGTTGTTGGATTCTAC |
| AgerOR26 | GAGAGCAAGTATTATTATCAAG | TTAGGCGATGGTTCTATA |
| AgerOR27 | ATACCCGCTCTATTACATTT | TTCACGACCAGGATTATG |
| AgerOR28 | ATCACAACGACTATACATACA | TAAGCAACAACTGGAACA |
| AgerOR29 | TTCATTAGAGATGTGGAGTTC | GCCTGATAGATTATTGTCGTA |
| AgerOR30 | CGATGAAGTAGAACGAAT | AACTGTTCAATGAGCATA |
| AgerOR31 | CTAAGCGAGGAAGATACATT | AAGCCGTTAGGTAGTGAT |
| AgerOR32 | GGTATGAACAATCGCTTTCTATA | GGCAATCCTTAATTCCAGAG |
| AgerOR33 | AGCATTCGCAATCTTATC | CAACACAGTTCATTAGCA |
| AgerOR34 | ACTCACCTTGTATTGTTG | CCTCATCATAGTTAGAATCAT |
| AgerOR35 | GCCATTGCTATTGAAGGT | CAGTAGAGCGAAGTAGGA |
| AgerOR36 | CACGACCACCTTCTTCTTC | TGCCGCACGAACTTATTG |
| AgerOR37 | CAGCAGTATTCTTCGTAA | GTCATCTAACAGCAGTAG |
| AgerOR38 | ATCTTATCTGCTGATGGAGTTG | CGGAGGCTTGAATTGCTAA |
| AgerOR39 | GTTGGTTACATATTCTACTAC | TGCTCTGTTCATAATCATA |
| AgerOR40 | CGGAGATGTCATATACGATACC | GCACCTCATCATTACGAAGA |
| AgerOR41 | TTATGTTGACCAGCCTAC | AACTTCGGAGAATGTATCA |
| AgerOR42 | GCAGATATGGTATATGATGAGA | TGTTGACAATGTGATAATTCG |
| IRs |  |  |
| AgerIR1 | TCTTAGTGGTCGTTGTAG | CTGAGTCTCTGAGTTCTG |
| AgerIR2 | AGGAGATTAAGAGGTGTA | GTTCTACTATGCCTGATT |
| AgerIR3 | CGTCTTCATAGTCATCTT | TACTTGTACCACCAATAC |
| Endogenous reference genes |  |  |
| Actin | CCTAAGTCGGCTATGTTATCG | GGTATCCTAATGTCGGTTCTTC |
